# Chronic Alcohol Exposure Disturbs Lipid Homeostasis at the Adipose Tissue-Liver Axis in Mice: Analysis of Triacylglycerols Using High-Resolution Mass Spectrometry in Combination with *In Vivo* Metabolite Deuterium Labeling

**DOI:** 10.1371/journal.pone.0055382

**Published:** 2013-02-06

**Authors:** Xiaoli Wei, Xue Shi, Wei Zhong, Yantao Zhao, Yunan Tang, Wenlong Sun, Xinmin Yin, Bogdan Bogdanov, Seongho Kim, Craig McClain, Zhanxiang Zhou, Xiang Zhang

**Affiliations:** 1 Chemistry Department, University of Louisville, Louisville, Kentucky, United States of America; 2 Medicine Department, University of Louisville, Louisville, Kentucky, United States of America; 3 Bioinformatics and Biostatistics Department, University of Louisville, Louisville, Kentucky, United States of America; 4 Pharmacology & Toxicology Department, University of Louisville, Louisville, Kentucky, United States of America; 5 Alcohol Research Center, University of Louisville, Louisville, Kentucky, United States of America; 6 Robley Rex Louisville VAMC, Louisville, Kentucky, United States of America; 7 Center for Translational Biomedical Research and Department of Nutrition, University of North Carolina at Greensboro, North Carolina Research Campus, Kannapolis, North Carolina, United States of America; National Institutes of Health, United States of America

## Abstract

A method of employing high-resolution mass spectrometry in combination with *in vivo* metabolite deuterium labeling was developed in this study to investigate the effects of alcohol exposure on lipid homeostasis at the white adipose tissue (WAT)-liver axis in a mouse model of alcoholic fatty liver. In order to differentiate the liver lipids synthesized from the fatty acids that were transported back from adipose tissue and the lipids synthesized from other sources of fatty acids, a two-stage mouse feeding experiment was performed to incorporate deuterium into metabolites. Hepatic lipids extracted from mouse liver, epididymal white adipose tissue (eWAT) and subcutaneous white adipose tissue (sWAT) were analyzed. It was found that 13 and 10 triacylglycerols (TGs) incorporated with a certain number of deuterium were significantly increased in alcohol induced fatty liver at two and four weeks of alcohol feeding periods, respectively. The concentration changes of these TGs ranged from 1.7 to 6.3-fold increase. A total of 14 deuterated TGs were significantly decreased in both eWAT and sWAT at the two and four weeks and the fold-change ranged from 0.19 to 0.77. The increase of deuterium incorporated TGs in alcohol-induced fatty liver and their decrease in both eWAT and sWAT indicate that alcohol exposure induces hepatic influx of fatty acids which are released from WATs. The results of time course analysis further indicate a mechanistic link between adipose fat loss and hepatic fat gain in alcoholic fatty liver.

## Introduction

It has been understood that dietary fats are digested in the intestinal epithelial cells, and then converted to triacylglycerols (TGs). TGs are assembled with apolipoproteins to form chylomicrons which are transported into the blood stream *via* the lymph system [1]. TGs are also synthesized by the liver where they are packaged as very low-density lipoproteins (VLDL) and secreted into the blood [1]. Upon arrival in the adipose and muscle tissues, lipoprotein lipase cleaves TG into free fatty acids and glycerol. Fatty acids are taken up by these tissues, and are used as energy sources *via* oxidation in muscles, or re-assembled into TGs to store excess energy in the white adipose tissue (WAT) [2]. Glycerol is transported to liver or kidneys where it is converted into dihydroxyacetone phosphate by glycerol kinase and glycerol-3-phosphate dehydrogenase.

WAT plays an important role in regulation of whole body energy homeostasis. WAT stores excess energy in the form of TG under positive energy balance condition, and releases fatty acids for energy generation under negative energy balance condition [2]. However, excess fatty acid release from the WAT may cause fatty acid overflux into the liver, leading to development of fatty liver [3]. Fatty liver is frequently associated with both alcohol abuse (alcoholic fatty liver, AFL) and obesity (nonalcoholic fatty liver, NAFL). Although previous studies have demonstrated similarities and differences in the pathogenesis of fatty liver between alcohol abuse and obesity, increased fatty acid uptake has been suggested to be a common mechanism for AFL and NAFL [4], [5]. While hepatocytes isolated from both alcohol-fed and obese rats showed an increased fatty acid uptake [6], further investigation is needed to provide direct evidence that fatty acids released from the WAT are indeed deposited in the liver.

Clinical studies have demonstrated that lower fat mass (lipodystrophy) was associated with higher liver fat in alcoholics [7], [8]. Animal models of AFL also showed that reduction of WAT mass was associated with an increased fatty acid uptake by hepatocytes [9]–[11]. Our study demonstrated that alcohol exposure to mice caused more hepatic accumulation of TGs which were labeled before alcohol exposure [12]. We also found that alcohol exposure stimulated adipose lipolysis and fatty acid release from WAT [12], [13]. These data suggest that alcohol exposure may cause an excess reverse fatty acid transport, thereby inducing fatty liver. Therefore, the animal model of AFL could be an ideal model to identify the importance of WAT in maintaining lipid homeostasis at the WAT-liver axis. Further determination of triacylglycerol homeostasis at the WAT-liver axis could reveal the direct link between WAT and liver, an organ-organ interaction mechanism, in the development of fatty liver.

The objective of this work was to use high-resolution mass spectrometry in combination with metabolite deuterium labeling to test our hypothesis that alcohol exposure disturbs lipid homeostasis at the WAT-liver axis towards triacylglycerol epitomic deposition in the liver. In order to differentiate the liver lipids synthesized using fatty acids from other sources from that synthesized using the fatty acids transported back from adipose tissue, a two-stage feeding experiment was performed, where all mice were first fed with deuterated water (^2^H_2_O) to ensure that the lipids stored in adipose tissue are deuterium labeled (stage one). The mice were then randomly grouped into two cohorts, the control cohort and the test cohort. Mice in the test cohort were fed an alcohol-containing liquid diet while mice in the control cohort were pair-fed an isocaloric maltose dextrin control liquid diet. The mice in both the control and test cohorts were then sacrificed at different times (stage two). Metabolite extracts from mouse liver, epididymal white adipose tissue (eWAT) and subcutaneous white adipose tissue (sWAT) were analyzed using linear trap quadrupole–Fourier transform ion cyclotron resonance mass spectrometer (LTQ-FTICR MS) *via* direct infusion electrospray ionization–mass spectrometry.

## Methods

### Animals and Treatments

Male C57BL/6N mice were obtained from Harlan (Indianapolis, IN, USA). All the mice were treated according to the experimental procedures approved by the University of Louisville Animal Care and Use Committee. To label lipids in adipose tissues, an approach using ^2^H_2_O as the metabolic tracer was followed [14]. Mice at two months old were given an initial priming dose of 99.8% ^2^H_2_O *via* an intraperitoneal injection to achieve 2.5% of body water enrichment, followed by administration of 5% ^2^H_2_O in the drinking water for five weeks (stage one, time point 0 week). The mice were then randomly grouped into two cohorts, the control cohort and the test cohort, for a 4-week of alcohol exposure (stage two). The test cohort was fed a modified Lieber-DeCarli alcohol liquid diet which contained 1,000 kcal/L calories, 34% from alcohol, 18% from protein, 34% from fat, and 14% from carbohydrate. The control cohort was fed a modified Lieber-DeCarli control liquid diet which also contained 1,000 kcal/L calories with replacement of the alcohol calories by isocaloric maltose dextrin. The alcohol-fed mice were free access to the alcohol diet, while the pair-fed mice were given the control diet in the same amount consumed by alcohol-fed mice in the previous day. The liquid diet feeding was conducted for 2 (time point of two weeks) or 4 (time point of four weeks) weeks. At the end of each feeding time point, mice were anesthetized, and liver, eWAT and sWAT tissues were collected from each mouse for measuring lipid components labeled by deuterium. There were 5, 5, 3 and 5, 7, 5 mice at time point 0, 2, 4 weeks for the control cohort and the test cohort, respectively.

### Tissue Sample Preparation

Liver, eWAT and sWAT samples were weighed, homogenized for 2 min and stored at –80°C until use. To extract metabolites from the homogenized tissue, 100 µL of homogenized tissue sample, 20 µL of butylatedhydroxytoluene (BHT) mixture (50 mg BHT into 1 mL methanol) and 2.0 mL chloroform–methanol (*v/v* = 2∶1) were mixed and vortexed for 2 min followed by adding 420 µL of water and vortexing for 2 min. The mixture was then centrifuged at room temperature at 2,000 rpm for 8 min. 400 µL of the organic phase (bottom) was aspirated into another glass tube and dried using a nitrogen evaporator. The dried sample was then dissolved into 200 µL of chloroform–methanol (*v/v* = 2∶1).

### FT-MS and LTQ-MS/MS Analysis

The direct infusion experiments were performed on a hybrid mass spectrometer, the so-called linear trap quadrupole – Fourier transform ion cyclotron resonance mass spectrometer (LTQ-FTICR MS or LTQ-FT MS) (Thermo Electron Corporation, Bremen, Germany) equipped with a chip-based nano-electrospray ionization (nESI) ion source (TriversaNanoMate) (Advion Biosciences, Ithaca, NY, USA). The mass spectrometer was operated in the positive ion mode. Each metabolite extract was measured for 5 min covering the *m/z* = 100−1,600 range. The mass spectra were recorded using the FTICR in profile mode and the resolving power (RP) was set at 400,000 @ *m/z* = 400. The maximum ion accumulation time was set at 1,000 ms. The ion optics was tuned for the sodium adduct of tricaprylin ([C_27_H_50_O_6_+Na^+^]) at *m/z* = 493.25 using the linear ion trap (LIT). The two most important nESI parameters were as follows: the spray voltage = +1.8 kV and the nitrogen gas pressure = 0.5 psi. The MS/MS spectrum of each metabolite ion was acquired on the LTQ. The parameters were set as follows: precursor ion *m/z* isolation window = ±0.3, spectrum accumulation time = 1 min. The normalized collision energy (NCE) is a molecule dependent parameter and ranged from 16 to 40%.

### Metabolite Quantification

The experimental data were processed using software package *MetSign* [15]. After peak alignment, a contrast based method was employed for normalization [16], [17]. Both the Fisher’s exact test and the pairwise two-tail t-test were used to study the concentration change of each metabolite between the two physiological conditions. The parameters used during the analysis are as follows: precursor ion *m/z* accuracy ≤5 ppm and the q-value for false discovery rate (FDR) ≤0.2 [18]. Temporal analysis was performed to study the correlation between time course trajectories measured by Pearson’s correlation coefficient and the distance measured by Fisher’s combined probability test [19], [20].

### Metabolite Identification

Metabolite identification was achieved in two sequential steps, database search and MS/MS characterization. Such a metabolite identification process meets the requirement of Level 2 metabolite identification, *i.e*., putatively annotated metabolites [21]. The metabolite database search was accomplished by the *MetSign* software using the FTICR-MS data. Each of the measured metabolite ion *m/z* value and isotopic peak profile were compared to the corresponding theoretical information of metabolites recorded in the *MetSign* database, which was composed of all metabolites recorded in the Kyoto Encyclopedia of Genes and Genomes, LIPID MAPS, and the Human Metabolome Database, resulting in 43,245 records. Possible positive-mode adduct ions include H^+^, Na^+^, K^+^, and NH_4_^+^.

To narrow down the metabolite candidates generated by database searching, the MS/MS spectra of metabolite peaks with significant concentration changes between two sample cohorts were acquired on LTQ-MS/MS. Each experimental MS/MS spectrum was compared to the *in silico* MS/MS spectra of all metabolite candidates using *Mass Frontier* 6.0 (Thermo Scientific, FL, USA). The spectral similarity between the experimental MS/MS spectrum and the *in silico* MS/MS spectrum of the metabolite of interest was evaluated using Pearson’s correlation coefficient. The metabolite candidate(s) with the best MS/MS spectrum match was (were) considered as the metabolite giving rise to the experimental spectrum.

### Measurements of Liver Steatosis and Routine Parameters

Neutral lipids in the liver were detected by Oil red O staining. Liver cryostat sections were cut at 7 µM, fixed with 10% formalin for 5 min, and stained with Oil red O in 2-propynal solution for 10 min. Plasma alanine aminotransferase (ALT) activity and triglyceride and cholesterol concentrations were determined using Infinity Reagents (Thermo Scientific, Middletown, VA). Plasma free fatty acids (FFA) were quantified using a FFA Quantification Kit (BioVision, San Francisco, CA). Statistical differences were analyzed by one-way ANOVA followed by Bonferroni *post hoc* comparison. The data are presented as mean ± SD and *p* values less than 0.05 were considered as significant.

## Results

### Metabolite Identification

**Figure 1:**
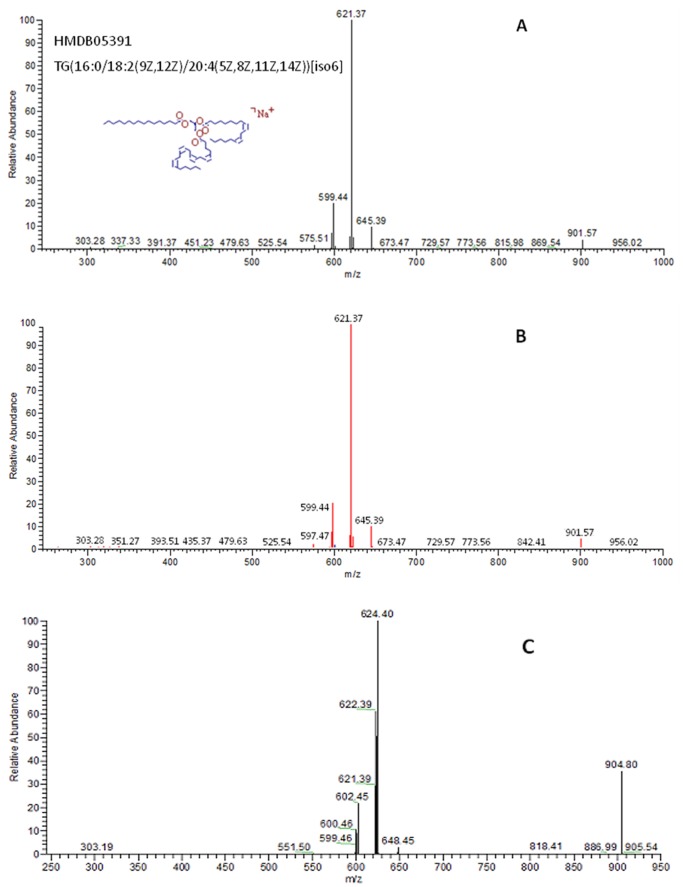
An example of identifying a deuterium incorporated metabolite using MS/MS information. The metabolite ion *m/z* value was measured on FTICR-MS as 904.74066. (A) is the experimental MS/MS spectrum of non-deuterated metabolite. (B) is the matching result of the non-deuterated metabolite between the experiment MS/MS spectrum and the theoretical MS/MS spectrum generated by *Mass Frontier*. The matched fragment ions are highlighted in red and the not matched ions in black. (C) is the MS/MS spectrum of deuterium incorporated metabolite.

The metabolite initial assignment *via* database search was achieved using high-resolution FTICR-MS data. Figure 1 is a sample of putative identification of triacylglycerol TG(16∶0/18∶2/20∶4)[iso6]. The *m/z* value of this metabolite was measured as 904.7407 by FTICR-MS. By searching the 43,245 database metabolites, this metabolite ion and its isotopic peak profile match the corresponding theoretical information of metabolites HMDB05391, HMDB10508, and HMDB13423 with adducts of Na^+^, H^+^ and K^+^, respectively. The number of deuterium atoms incorporated in these three metabolite candidates is 3, 5, and 6, respectively. Therefore, the *m/z* values of the corresponding non-deuterium incorporated metabolites of these three metabolite candidates should be 901.7407, 899.7407 and 898.7407, respectively. In order to confirm the initial assignment, LTQ-MS/MS experiments were performed to acquire MS/MS spectra for each of these metabolite ions. Figure 1A is the LTQ-MS/MS spectrum of the metabolite ion with a measured *m/z* = 901.57. The molecular structures of the candidate metabolites HMDB05391, HMDB10508, and HMDB13423 were then uploaded into *Mass Frontier* with corresponding adducts Na^+^, H^+^ and K^+^, respectively, to generate *in silico* MS/MS spectra for each of these three candidates. Each of the *in silico* spectra was then matched to the experimental LTQ-MS/MS spectrum. The *in silico* MS/MS spectrum of metabolite HMDB05391+Na^+^ has the best match with a Pearson’s correlation coefficient of 0.9981 and therefore, this metabolite was considered as the metabolite present in the sample (Figure 1B).

It should be pointed out that *Mass Frontier* software can only predict the *m/z* values of fragment ions, but not the fragment ion abundance. It then matches the *m/z* values of the predicted fragment ions to the *m/z* values of experiment mass spectrum. Therefore, a high value of Pearson’s correlation coefficient only refers to the matching quality of fragment ion *m/z* values between an *in silico* MS/MS and an experiment MS/MS spectrum.

The incorporated deuterium atoms in a metabolite do not significantly affect the metabolite fragmentation during MS/MS analysis, resulting in similar MS/MS spectra between the deuterium incorporated metabolite and the corresponding non-deuterium incorporated metabolite. The only difference between the MS/MS spectra is *m/z* values of the fragment ions that carry deuterium atoms. The difference in the *m/z* values between the corresponding fragment ions in the two spectra may range from zero to the mass of all incorporated deuterium atoms. Figure 1C is the LTQ-MS/MS spectrum of a deuterium incorporated version of metabolite HMDB05391 with a measured metabolite ion *m/z* = 904.80. The corresponding fragment ions between the deuterium incorporated fragment ions and the non-deuterium incorporated fragment ions are (904.80, 901.57), (887.99, 883.62), (648.45, 645.39), (624.40, 621.37), (622.39, 619.44), (602.45, 599.44) and (600.46, 597.47). The number of incorporated deuterium atoms in each fragment ion is 3, 3, 3, 3, 3, 3 and 3, respectively. The spectral similarity between the spectrum of a deuterium incorporated metabolite (Figure 1C) and the spectrum of the corresponding non-deuterium incorporated metabolite (Figure 1A) was evaluated using Pearson’s correlation coefficient, after recognizing the pairs of fragment ions between the spectrum of a deuterium incorporated metabolite and the spectrum of a non-deuterium incorporated metabolite. The Pearson’s correlation coefficient between the top 10 abundant fragment ions in the two spectra displayed in Figures 1A and 1C is 0.8744, showing the high similarity between these two spectra.

### Statistical Significance Tests

**Figure 2:**
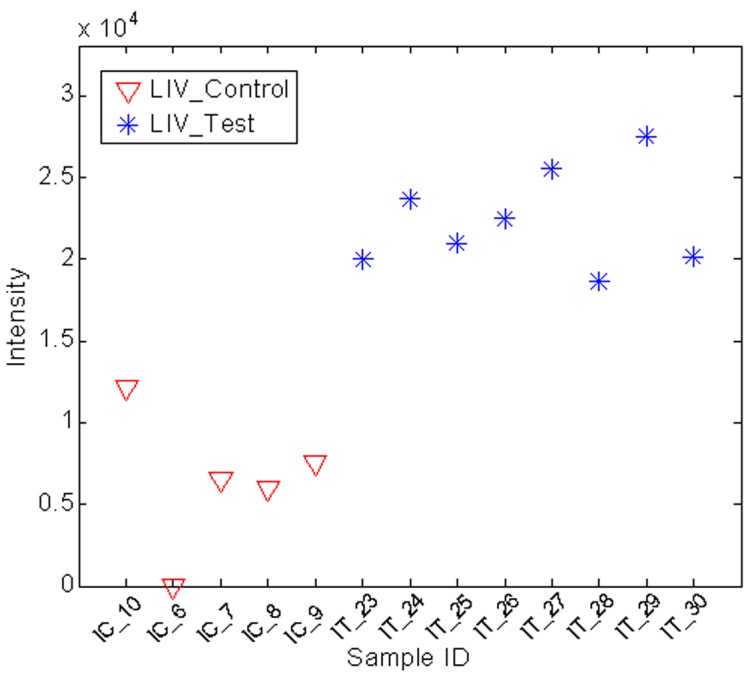
Sample concentration changes of metabolite in two different physiological conditions. The abundance test (pair-wise two-tail t-test) shows that the concentration of this metabolite in the test cohort is increased with a fold change of 2.8 and a p-value of 1.4×10^−5^. This metabolite was further identified as TG(16∶0/18∶2/20∶4)[iso6] by MS/MS analysis.

Figure 2 depicts peak area distribution of triacylglycerol TG(16∶0/18∶2/20∶4)[iso6] at time two weeks among the samples of the test and control cohorts. It can be seen that this molecule is significantly increased with a 2.8-fold change in the test cohort compared to its level in the control cohort. The fold change was defined as the ratio of the average peak area of a metabolite measured in the test cohort divided by the average peak area of the same metabolite in the control cohort. The p-value of the pairwise two-tail t-test is 1.4×10^−5^.

Table 1 lists all of the metabolites identified with significant concentration changes in liver between the control cohort and the test cohort at two and four weeks. All of these metabolites were identified as deuterium incorporated TGs even though all the 43,245 metabolites were searched for the metabolite identification. Secondly, the deuterium incorporated TGs, 13 at 2 weeks and 10 at 4 weeks, were all increased in the test cohort with a 1.7 to 6.3-fold change. Tables 2 list all of the metabolites identified with significant concentration changes between the control cohort and the test cohort at two and four weeks in the eWAT and sWAT. These metabolites are all deuterium labeled and identified as TGs. The number of deuterated TGs was more in the eWAT (10 TGs) compared to the sWAT (4 TGs). All these deuteraed TGs were reduced by alcohol exposure at either two weeks or four weeks with a fold-change ranged from 0.19 to 0.77.

**Table 1:** List of triacylglycerols in liver identified with significant concentration changes between the control cohort and the test cohort at two and four weeks.

Time (Week)	*m/z*	p-value	Fold change (T/C)^a^	Metabolite common name	Adduct ion	No. ^2^H
2	878.7338	7.8×10^−3^	2.1	TG(16∶1/18∶2/20∶4)[iso6]	H^+^	1
2	902.7352	1.0×10^−4^	8.1	TG(16∶0/18∶2/20∶4)[iso6]	Na^+^	1
2	904.7407	1.4×10^−5^	2.8	TG(16∶0/18∶2/20∶4)[iso6]	Na^+^	3
2	907.7698	2.8×10^−3^	2.8	TG(16∶0/18∶0/20∶4)[iso6]	Na^+^	4
				TG(16∶0/20∶4/20∶4)[iso3]	H^+^	4
2	926.7360	6.9×10^−3^	4.2	TG(16∶0/20∶4/20∶4)[iso3]	Na^+^	1
				TG(18∶3/18∶2/22∶6)[iso6]	H^+^	1
2	927.7376	2.3×10^−3^	1.9	TG(16∶0/20∶4/20∶4)[iso3]	Na^+^	2
2	928.7407	5.7×10^−3^	1.9	TG(16∶0/20∶4/20∶4)[iso3]	Na^+^	3
2	929.7534	1.6×10^−2^	1.7	TG(16∶0/20∶4/20∶4)[iso3]	Na^+^	4
				TG(18∶3/18∶2/22∶6)[iso6]	H^+^	4
2	930.7666	3.1×10^−2^	3.1	TG(18∶3/18∶3/20∶0)[iso3]	Na^+^	1
2	952.7521	8.6×10^−3^	6.3	TG(18∶1/20∶4/20∶4)[iso3]	Na^+^	1
				TG(18∶3/20∶4/22∶6)[iso6]	H^+^	3
2	954.7666	9.1×10^−3^	3.0	TG(20∶4/18∶1/22∶6)[iso6]	H^+^	1
2	903.7797	7.2×10^−3^	na b	TG(16∶0/18∶0/18∶0)[iso3]	K^+^	2
2	905.7533	4.8×10^−3^	na^b^	TG(16∶0/18∶2/20∶4)[iso6]	Na^+^	4
				TG(16∶0/20∶4/20∶4)[iso3]	H^+^	2
4	902.7352	2.1×10^−3^	3.8	TG(16∶0/18∶2/20∶4)[iso6]	Na^+^	1
4	904.7407	4.4×10^−2^	1.7	TG(16∶0/18∶2/20∶4)[iso6]	Na^+^	3
4	926.7360	1.8×10^−3^	2.7	TG(16∶0/20∶4/20∶4)[iso3]	Na^+^	1
				TG(18∶3/18∶2/22∶6)[iso6]	H^+^	1
4	927.7376	3.8×10^−3^	2.5	TG(16∶0/20∶4/20∶4)[iso3]	Na^+^	2
4	928.7407	3.6×10^−2^	1.7	TG(16∶0/20∶4/20∶4)[iso3]	Na^+^	3
4	929.7534	2.2×10^−2^	1.9	TG(16∶0/20∶4/20∶4)[iso3]	Na^+^	4
				TG(18∶3/18∶2/22∶6)[iso6]	H^+^	4
4	930.7666	3.2×10^−2^	2.8	TG(18∶3/18∶3/20∶0)[iso3]	Na^+^	1
4	952.7521	2.8×10^−2^	3.6	TG(18∶1/20∶4/20∶4)[iso3]	Na^+^	1
				TG(18∶3/20∶4/22∶6)[iso6]	H^+^	3
4	953.7545	2.5×10^−2^	2.7	TG(18∶3/20∶4/22∶6)[iso6]	H^+^	4
4	954.7666	5.3×10^−3^	3.3	TG(20∶4/18∶1/22∶6)[iso6]	H^+^	1

a Fold-change is the ratio of average peak area of a metabolite in the test cohort (T) to that in the control cohort (C).

b na refers to a metabolite that was detected only in the test cohort. Therefore, the values of fold change for these metabolites are not available.

**Table 2:** List of triacylglycerols in sWAT and eWAT identified with significant concentration changes between the control cohort and the test cohort at two and four weeks.

WAT	Time (Week)	*m/z*	p-value	Fold change (T/C)^a^	Metabolite common name	Adduct ion	No. ^2^H
sWAT	2	879.7446	2.1×10^−2^	0.74	TG(16∶1/18∶2/20∶4)[iso6]	H^+^	2
	2	880.7507	3.9×10^−2^	0.69	TG(16∶0/18∶1/18∶2)[iso6]	Na^+^	1
	4	826.7013	1.7×10^−3^	0.28	TG(16∶0/16∶1/16∶1)[iso3]	Na^+^	1
	4	879.7446	1.5×10^−2^	0.74	TG(16∶1/18∶2/20∶4)[iso6]	H^+^	2
eWAT	2	826.7017	4.1×10^−4^	0.38	TG(16∶0/16∶1/16∶1)[iso3]	Na^+^	1
	2	850.7013	1.3×10^−2^	0.68	TG(16∶1/16∶1/18∶2)[iso3]	Na^+^	1
	2	854.7334	2.7×10^−3^	0.58	TG(16∶0/16∶1/20∶4)[iso6]	H^+^	1
	2	855.7378	1.9×10^−2^	0.70	TG(16∶0/16∶0/18∶2)[iso3]	Na^+^	2
	4	828.7174	1.6×10^−2^	0.19	TG(16∶0/16∶1/16∶1)[iso3]	Na^+^	3
	4	854.7334	1.5×10^−2^	0.54	TG(16∶0/16∶1/20∶4)[iso6]	H^+^	1
	4	855.7378	4.4×10^−2^	0.48	TG(16∶0/16∶0/18∶2)[iso3]	Na^+^	2
	4	856.7482	2.2×10^−3^	0.49	TG(16∶0/16∶0/18∶1)[iso3]	Na^+^	1
	4	879.7445	1.5×10^−2^	0.73	TG(16∶1/18∶2/20∶4)[iso6]	H^+^	2
	4	880.7498	7.7×10^−3^	0.77	TG(16∶0/18∶1/18∶2)[iso6]	Na^+^	1

a Fold-change is the ratio of average peak area of a metabolite in the test cohort (T) to that in the control cohort (C).

### Temporal Analysis

**Figure 3:**
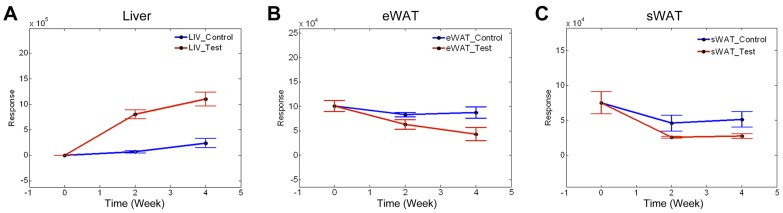
Sample time course trajectories of deuterium labeled triacylglycerols detected in liver, eWAT and sWAT samples. (A) TG(16∶0/18∶2/20∶4)[iso6] with one ^2^H label and one Na^+^ as adduct ion in liver samples. (B) TG(16∶0/16∶1/16∶1)[iso3] with one ^2^H label and an adduct ion of Na^+^ eWAT samples. (C) TG(16∶0/16∶0/18∶1)[iso3] with one ^2^H label and an adduct ion of Na^+^ in sWAT samples.

Even though the statistical significance tests support the hypothesis of reverse fatty acid transport, it is still necessary to investigate the trajectory of each metabolite in the time course. Figure 3 shows three sample time course trajectories in liver, eWAT and sWAT, respectively. Figure 3A displays the time course trajectory of triacylglycerol TG(16∶0/18∶2/20∶4)[iso6] with one ^2^H label and one Na^+^ as adduct ion in liver samples. While this TG molecule was not significantly increased in control cohort, it was significantly increased in test cohort at 2 weeks and a further elevation was found at 4 weeks. Figures 3B and 3C show the time course trajectories of TG(16∶0/16∶1/16∶1)[iso3] with one ^2^H label and an adduct ion of Na^+^ in eWAT samples, TG(16∶0/16∶0/18∶1)[iso3] with one ^2^H label and an adduct ion of Na^+^ in sWAT samples, respectively. In contrast to the increase in the liver of test cohort, a decrease in deuterium-labeled TGs was observed in both the eWAT and sWAT of test cohorts. The deuterium-labeled TG(16∶0/16∶1/16∶1)[iso3] in eWAT significantly declined at 2 weeks and a further decrease was found at 4 weeks. While deuterium-labeled TG(16∶0/16∶0/18∶1)[iso3] in sWAT significantly declined at 2 weeks, no further decrease was found at 4 weeks. Time course changes for other deuterium-labeled TGs in liver and WAT were listed in Table 1 and Table 2, respectively.

**Figure 4:**
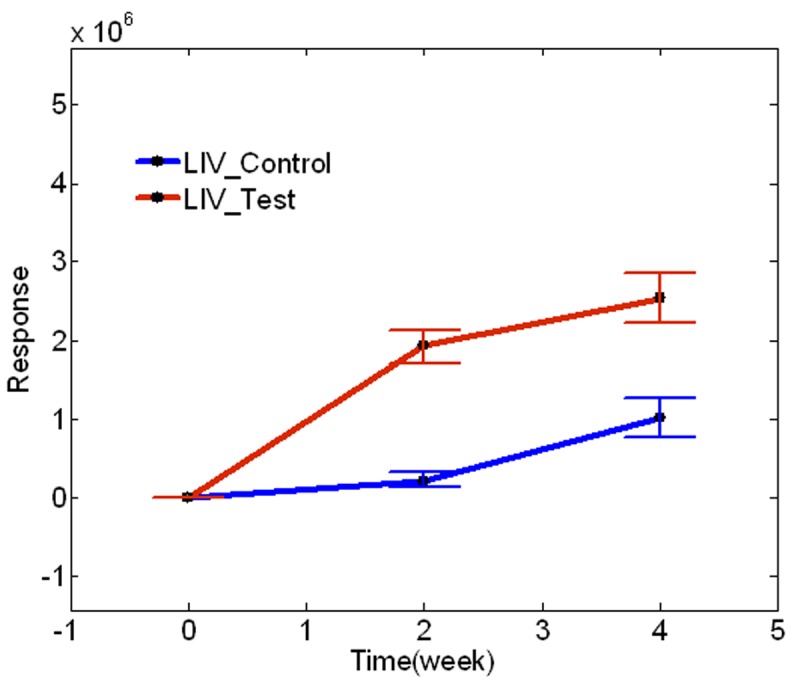
Time course trajectory of triacylglycerol TG(16∶0/18∶2/20∶4)[iso6] without any deuterium labeling in liver samples.

Figure 4 shows that the time course trajectory of triacylglycerol TG(16∶0/18∶2/20∶4)[iso6] without ^2^H label in liver samples of control cohort and test cohort. This TG molecule represents hepatic TGs which are synthesized from the dietary fats or from *de novo* lipogenesis, because it did not incorporate any deuterium. In the liver of control cohort, this TG molecule did not change at 2 weeks but increased at 4 weeks. Surprisingly, the test cohort showed a remarkable increase at 2 weeks compared to time 0, and a further increase at 4 weeks. The abundance of this TG molecule in the test cohort was significantly higher than that in the control cohort at both 2 and 4 weeks.

### Alternations of Hepatic Neutral Lipid and WAT Mass

**Figure 5:**
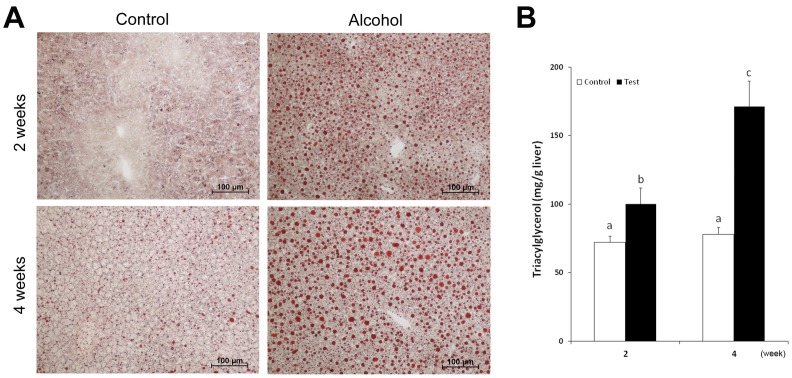
Time course changes of hepatic lipid content. (A) Hepatic neutral lipid detected by Oil red O staining of cryostat liver sections. Alcohol exposure increased hepatic neutral lipid (lipid droplets) gradually along the 4 weeks of experiment. (B) Hepatic TG concentrations were quantitatively measured. Data are expressed as mean ± SD (*n* = 6−8). Statistical differences were analyzed by ANOVA followed by Bonferroni *post hoc* comparison, and means without a common letter differ at *p*<0.05.

**Figure 6:**
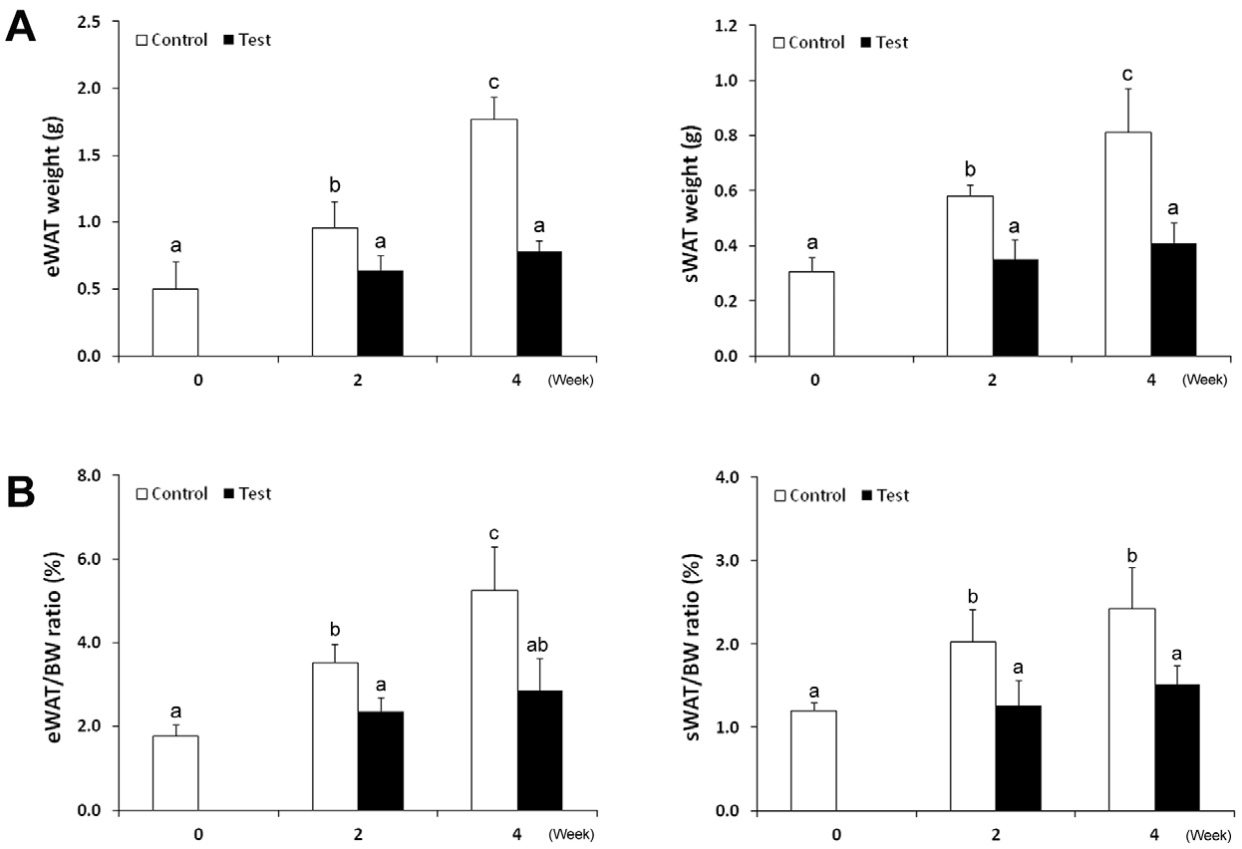
Time course changes of WAT tissues. (A) WAT mass. The weights of both eWAT and sWAT in control mice increased gradually during the 4 weeks of experiment. However, the alcohol-fed mice did not show weight change in both eWAT and sWAT at either 2 weeks or 4 weeks. (B) WAT to body weight ratio (%). Data are expressed as mean ± SD (*n* = 6−8). Statistical differences were analyzed by ANOVA followed by Bonferroni *post hoc* comparison, and means without a common letter differ at *p*<0.05.

To determine an overall change in lipid homeostasis at the liver-WAT axis, neutral lipid in the liver and WAT mass were measured. As shown in Figure 5, oil red O staining of neutral lipid on cryostat liver sections clearly demonstrated accumulation of lipid droplets in the hepatocytes of alcohol-fed mice at 2 weeks. Further increases in number and size of the lipid droplets were observed at 4 weeks. In contrast to neutral lipid accumulation in the liver, WAT mass was significantly lower in the alcohol-fed mice compared to the controls (Figure 6). The weights of both eWAT and sWAT from control mice were increased at 2 weeks and a further increase was found at 4 weeks. However, the weights of both eWAT and sWAT from alcohol-fed mice did not change at either 2 weeks or 4 weeks, leading to an increased difference between the control and alcohol mice along the 4 weeks of experiment. The time course changes in WAT to body weight ratio showed similar trends to that of WAT mass.

### Routine Parameters

Table 3 listed the results of routine parameters including body weight, liver weight, liver to body weight ratio, and plasma ALT activity and FFA concentration. The test cohort showed a lower body weight but a higher liver weight, leading to a significant increase in liver/body weight ratio at both 2- and 4-week time points. The plasma ALT activity level, an indicator of liver injury, was elevated in the test cohort at both time points. The plasma triacylglycerol level was also increased in the test cohort at 4-week. However, plasma cholesterol and FFAs was not affected by alcohol exposure.

**Table 3:** Body weight, liver weight and plasma parameters of the control cohort and the test cohort at two and four weeks.

	2 weeks		4 weeks	
	Control	Test	Control	Test
Body weight(BW, g)	28.6±0.9 a	26.9±1.3 a	32.4±2.7 b	27.9±1.2 a
Liver weight (g)	1.06±0.04 a	1.16±0.05 b	1.10±0.04 ab	1.32±0.08 c
Liver/BW ratio (%)	3.69±0.22 a	4.33±0.30 b	3.41±0.16 a	4.77±0.21 c
ALT (U/L)	20.4±9.1 a	48.2±9.1 b	25.6±3.1 a	58.6±12.6 b
Triacylglycerol(mg/dL)	93.2±10.4 a	130.5±24.7 ab	110.4±11.3 a	171.9±47.7 b
Cholesterol(mg/dL)	121.7±19.6	107.1±14.8	110.7±10.3	101.4±10.3
FFA (mg/dL)	0.36±0.02	0.31±0.05	0.34±0.05	0.33±0.07

Data are expressed as mean ± SD (*n* = 6−8). Statistical differences were analyzed by ANOVA followed by Bonferroni *post hoc* comparison, and means without a common letter differ at *p*<0.05.

## Discussion

TGs are the group of most abundant lipids in liver, eWAT and sWAT. Analysis of the abundance changes of TGs is enough for us to test our hypothesis. Therefore, the methanol/water phase of metabolite extract from mouse tissues was discarded during the process of metabolite extraction, while the organic phase was used for analysis. It is possible that the regulations of other types of metabolites are also changed besides TGs during the mouse feeding period. However, the changes of these metabolites are not in the scope of this study.

### Biological Experiment Design

A two-stage animal feeding experiment was performed in this study to differentiate the hepatic lipids synthesized from the fatty acids transported back from adipose tissue from that synthesized using fatty acids from *de novo* lipogenesis. It is expected that majority of the lipids synthesized in the stage-one experiment were incorporated with a certain number of deuterium atoms, and most of them were transported and stored in WAT. The purpose of the stage-two experiment was to induce fatty liver in the test cohort and to use the mice in the control cohort as reference to monitor the lipid concentration change in the mice of the test cohort.

During the stage-two experiment, lipids were continuously synthesized in the mouse livers in both the test and the control cohorts. The lipids synthesized from the uptake of dietary fats should not incorporate any deuterium atoms, except a very small fraction of naturally occurring deuterium in the dietary fats. Therefore, two forms of lipids should be present in mouse liver: deuterium incorporated lipids and non-deuterium incorporated lipids. In case of the control cohort, the deuterium incorporated lipids were synthesized during the stage-one experiment while the non-deuterium incorporated lipids were synthesized in the stage-two experiment.

### Statistical Significance Tests

Compared to the levels of deuterium incorporated TGs in the control cohort, the significant increase of the deuterium incorporated TGs in the test cohort of liver samples (Table 1) indicates that extra deuterium incorporated fatty acids were used for the synthesis of TGs in alcoholic fatty liver at 2 and 4 weeks. The only source of the extra deuterium incorporated fatty acids is the WATs, where the deuterium incorporated fatty acids were stored in the form of TGs during the stage-one experiment. Therefore, a reasonable explanation to the increase of deuterium incorporated TGs in alcoholic fatty liver in the test cohort is that the deuterium incorporated fatty acids were transported back from the WAT after lipolysis due to alcohol consumption. Indeed, such an explanation is further substantiated by the decrease of deuterated TGs in eWAT and sWAT, respectively (Table 2). This supports our hypothesis, *i.e.*, alcohol consumption stimulates lipolysis in the WAT of mice, leading to significant release of fatty acids, which are transported back and deposited in the liver for the synthesis of TGs.

### Temporal Analysis

Figures 3A demonstrates that portions of the accumulated TGs in fatty liver were synthesized using deuterium incorporated fatty acids that were transported back from the WAT due to lipolysis, while Figure 3B and 3C demonstrate a significant abundance decrease of the deuterated TGs in eWAT and sWAT, respectively. These results reveal a direct link between WAT fatty acid release and hepatic TG deposition in the development of alcoholic fatty liver. A previous study also demonstrated that diminishing lipid storage function in WAT by over-expressing leptin-receptor b (lpr-b) on the aP2-lpr-b promoter (aP2lepr-b transgene) in db/db mice attenuated obesity after high fat feeding [22]. However, the aP2lepr-b transgene significantly increased liver weight and triglyceride concentrations, and accelerated the development of diabetes. Therefore, WAT dysfunction in lipid storage could be an important determinant in the pathogenesis alcoholic or nonalcoholic fatty liver.

Figure 4 displays the time course trajectory of triacylglycerol TG(16∶0/18∶2/20∶4)[iso6] without any deuterium labeling in liver samples. This TG molecule is synthesized by using fatty acids from dietary source and/or hepatic *de novo* lipogenesis rather than by using fatty acids transported back from the WAT. The time dependent abundance of this metabolite in the test sample is always higher than its abundance in the control sample at both 2 weeks and 4 weeks. These data indicate that fatty acids from the WAT are not the sole source of TG synthesis, and fatty acids from diet and/or *de novo* synthesis also contribute to the development of alcoholic fatty liver.

### WAT Dysfunction and Fatty Liver

Time course changes in WAT weight (Figure 5) demonstrated that WAT mass in control mice significantly increased along the 4 weeks of feeding. Surprisingly, the WAT mass of alcohol fed mice did not changed at either 2 weeks or 4 weeks compared to time 0, indicating a loss of the lipid storage function. A previous study has reported that alcohol feeding to rats reduced total body fat content due to an increase of TG turnover in rats, as indicated by a 2.3-fold increase in TG degradation with no significant change in TG synthesis [9]. Our previous report also showed that alcohol exposure activates lipolysis pathways in WAT, thereby accelerating fatty acid release [12]. Adipose lipolysis is regulated positively by catecholamine and negatively by insulin [23]. Previous studies suggested that alcohol-increased lipolysis is most likely through disturbing insulin signaling rather than enhancing catecholamine-mediated lipolysis [9], [12], [24]. Insulin negatively regulates lipolysis, and hosphodiesterase 4 (PDE4) and activating protein phosphatase 1 (PP1) mediate insulin signaling via reducing cellular cAMP level and dephosphorylating hormone sensitive lipase (HSL), respectively [23]. Although the adipose PDE4 was not affected in the WAT of alcohol-fed rats [9], our previous study showed that PP1 protein level was reduced in the WAT of alcohol-fed mice [12]. We also found that chronic alcohol exposure up-regulated negative regulators of insulin signaling, including phosphatase and tensin homolog (PTEN) and suppressor of cytokine signaling 3 (SOC3). In addition to lipid storage dysfunction, alcohol exposure also inhibited expression and secretion of adipokines including adiponectin and leptin in WAT [25]–[27]. Both adiponectin and leptin critically modulate hepatic lipid metabolism toward reduction of lipid content in the liver. Normalizing plasma adiponectin or leptin level was associated with attenuation of alcoholic fatty liver [28]–[30]. Therefore, adipose tissue dysfunction may contribute to the development of alcoholic fatty liver by directly supplying fatty acids for hepatic TG synthesis or indirectly disturbing adipokine regulation of hepatic lipid metabolism.

### Alcohol-induced Hepatic Lipid Dyshomeostasis

Alcoholic exposure may disturb hepatic lipid metabolism in multiple pathways, including fatty acid uptake, fatty acid oxidation, *de novo* lipogenesis and lipid export [25], [31]. The present study demonstrated that alcohol exposure causes a reverse transport of TGs from WAT to the liver. Liver plays a central role in lipid metabolism, but it does not store lipid at physiological condition. Balance between TG synthesis and export is a key determent of hepatic lipid homeostasis [32], [33]. While fatty acids from either blood or *de novo* synthesis are converted to TGs which are exported to the blood in the form of very low density lipoproteins (VLDL) for use or storage by the peripheral organs. Even though alcohol induces hepatic influx of fatty acids, fatty liver should not be developed as long as the liver can efficiently secrete TGs into the blood. Therefore, impaired VLDL secretion should co-exist with hepatic fatty acid influx in the development of alcoholic fatty liver. The present study shows that the liver of alcohol-fed mice accumulated TGs synthesized by using fatty acids of both deuterium labeled from WAT source and non-deuterium labeled from dietary fats or *de novo* lipogenesis. These data suggest that alcohol blunted lipid export. The authors believe that the increased blood TG levels in the test cohort at 4 weeks may indicate an impaired TG uptake from VLDL in WATs, rather than an increased hepatic TG secretion. Indeed, our previous study demonstrated that alcohol exposure significantly reduced the rate of VLDL-TG secretion from the liver to the blood [11], [13]. Disruption of VLDL secretion is likely an important mechanism underlying alcoholic fatty liver, because improvement of VLDL secretion was associated with attenuation of alcoholic fatty liver by zinc, betaine or hepatocyte growth factor [11], [34], [35]. Further investigations are required to determine the mechanisms of how alcohol exposure suppresses lipid export function of the liver.

### Conclusions

We used an analytical method of employing high-resolution mass spectrometry in combination with metabolite deuterium labeling for the analysis of triacylglycerol. A two-stage mouse feeding schema was designed, where all mice were first fed with deuterated water to label WAT TGs (stage one), followed by pair-feeding an alcohol or isocaloric maltose dextrin control liquid diet for two or four weeks. Hepatic lipids extracted from the livers, eWAT and sWAT tissues were detected by FTICR–MS and LTQ-MS/MS. All observations in this study, including the increase of TGs in the test cohort of liver, the simultaneous decrease of TGs in the test cohort of eWAT and sWAT, and the agreement between the metabolomics data and the histological data demonstrate that chronic alcohol exposure disturbs lipid homeostasis at the adipose tissue-liver axis and therefore, support our hypothesis, that is, alcohol consumption stimulates lipolysis of the WAT and leads to an excess release of fatty acids which are transported to the liver and deposited as TGs. Furthermore, accumulation of TGs synthesized from fatty acids from dietary source or *de novo* lipogenesis also contributes to the pathogenesis of fatty liver.
